# Asylum Visiting Committees in Conference

**Published:** 1918-11-30

**Authors:** 


### Asylum Visiting Committees in Conference.

A conference of visiting committees of asylums, convened by Mr. Thos. Field, chairman of the Visiting Committee of the Bucks County Asylum, was held in the Guildhall, London, on the 29th ultimo, to discuss suggested amendments to the Lunacy Laws and to consider an agenda drafted in view of the recommendations contained in the fourth annual report of the Board of Control. Some 170 delegates were present, consisting of members of asylum visiting committees, medical superintendents, and clerks to visiting committees.

The chairman, Alderman Morgan Thomas, J.P., ex-Lord Mayor of Cardiff, chairman of the Cardiff City Mental Hospital, argued that certain provisions had not been made for dealing with incipient mental trouble. Why was it not possible to attach to hospitals departments dealing with mental trouble or nervous breakdown? Why should not those whose nerves were giving way be able to go to a clinic or hospital instead of having to bear the stigma of going to an asylum? The question of a deputation to the Board of Control was referred to a committee to report thereon, the committee to consist of nine voluntary laymen, members of asylum visiting committees, four medical superintendents, and three clerks to visiting committees. Mr. Charles Fitch, clerk to the City of London Asylum Visiting Committee, consented to act as secretary *pro tem.* The resolution by Mr. Thos. Field was also referred to the committee. Mr. Field's reasons for the proposals are as follows:

Members of visiting committees of asylums feel that there should be closer co-operation to secure uniformity of policy in the carrying out of their duties. Local conditions vary, but the policy could be practically the same. On their administrative duties amongst other matters for consideration were general management, rules, business method and organisation, appointments, salaries and emoluments, and the increasing claims of the National Asylum Workers' Union and other workers. At present visiting committees were often working at cross-purposes. The desirability of forming a Parliamentary Committee to watch legislation affecting asylums and to take any necessary action was specially important. Whether a Ministry of Health would absorb the Board of Control or no, they could not say, but in view of the bureaucratic tendency of modern legislation it behoved all local authorities, and especially asylum authorities who were closely supervised by the Government, to pool their forces to safeguard their statutory powers and their freedom of action. If visiting committees were going to sacrifice their time, they should at least feel assured that their discretion was not to be hampered at every turn by a Government Department. There is a strong desire for co-operation amongst asylum visiting committees. A good method of bringing this about would be to establish a National Council of Visiting Committees of Asylums in England and Wales. The committee, which he now moved for, should give this matter its deep consideration. The conference was adjourned until early in the New Year.

